# Treatment of Mandibular Condyle Fractures Using a Modified Transparotid Approach via the Parotid Mini-Incision: Experience with 31 Cases

**DOI:** 10.1371/journal.pone.0083525

**Published:** 2013-12-26

**Authors:** Jun Shi, Hao Yuan, Bing Xu

**Affiliations:** Department of Craniomaxillofacial Science, Shanghai 9th People’s Hospital, School of Dentistry, Shanghai Jiaotong University, Shanghai, China; Van Andel Institute, United States of America

## Abstract

Surgery for mandibular condyle fractures must allow direct vision of the fracture, reduce surgical trauma and achieve reduction and fixation while avoiding facial nerve injury. This prospective study was conducted to introduce a new surgical approach for open reduction and internal fixation of mandibular condyle fractures using a modified transparotid approach via the parotid mini-incision, and surgical outcomes were evaluated. The modified transparotid approach via the parotid mini-incision was applied and rigid internal fixation using a small titanium plate was carried out for 36 mandibular condyle fractures in 31 cases. Postoperative follow-up of patients ranged from 3 to 26 months; in the first 3 months after surgery, outcomes for all patients were analyzed by evaluating the degree of mouth opening, occlusal relationship, facial nerve function and results of imaging studies. The occlusal relationships were excellent in all patients and none had symptoms of intraoperative ipsilateral facial nerve injury. The mean degree of mouth opening was 4.0 (maximum 4.8 cm, minimum 3.0 cm). No mandibular deviations were noted in any patient during mouth opening. CT showed complete anatomical reduction of the mandibular condyle fracture in all patients. The modified transparotid approach via the smaller, easily concealed parotid mini-incision is minimally invasive and achieves anatomical reduction and rigid internal fixation with a simplified procedure that directly exposes the fracture site. Study results showed that this procedure is safe and feasible for treating mandibular condyle fracture, and offers a short operative path, protection of the facial nerve and satisfactory aesthetic outcomes.

## Introduction

The most common mandibular fracture is the condylar fracture, and condylar neck and subcondylar fractures account for 17.5% to 52% of all mandibular fractures [1], [2]. Factors that influence treatment decisions include age of the patient, whether the fracture is unilateral or bilateral, presence of other mandibular fractures, the level and displacement of the fracture, the presence of teeth and the degree to which occlusion is disturbed [1], [3]. However, controversy over how to treat mandibular condyle fractures—conservative closed reduction and functional therapy vs. open surgical reduction—has existed since the first mandibular condyle neck surgery was performed in 1925 [4]. The lack of consensus is largely because the surgery is complex and morbidity is high, including possible infection, injury to the facial nerve or blood vessels and scar formation [5]. If occlusion is not disturbed, or only minimally disturbed, conservative treatment may simply involve a soft diet for several weeks and regular check-ups or application of arch bars and elastic traction, especially in pediatric patients [6], [7]. Closed reduction with functional therapy after initial intermaxillary fixation is considered a safe treatment with few complications and no scarring [5]. Decisions about whether to use open vs. closed procedures depends on surgeons’ preferences and levels of skill and experience [8], as well as the location of the fracture site and the severity of displacement [5]. Generally, surgeons who prefer surgical treatment insist that only open reduction and internal fixation can prevent long-term effects such as shortening of the ramus, facial asymmetry, pain and arthrosis of the temporomandibular joint (TMJ) and impairment of mastication and articular function; also, rehabilitation is achieved more quickly, so TMJ and mastication function will return to normal in a shorter period of time [9].

When addressing open surgical treatment of mandibular condyle fractures, surgeons must resolve problems such as how to expose the fracture site sufficiently while reducing surgical trauma as much as possible, and how to achieve excellent reduction and fixation while avoiding injury of the facial nerve and its branches. A number of approaches are used to help address these problems. The transparotid approach is considered to be an easy way to gain direct access to fractures of the mandibular ramus and condylar neck, allowing proximal anatomical reduction to be manipulated and fixed with miniplates [10]. The plates can be well-adapted and screws can be placed at 90 degrees to the bony surface, which provides greater mechanical advantages than the traditional retromandibular approach. However, proponents of the *modified* retromandibular approach report it to be a simple, short surgical procedure compared to the *traditional* retromandibular approach [11]. It completely exposes the operative field, which aids reduction and fixation and substantially reduces risk to the facial nerve. Recently, use of the retromandibular transparotid approach in treating 28 patients with condylar neck or condylar base fractures was described as having a short access route, easy reduction, short operating time and stable postoperative occlusion without permanent damage from facial nerve injury, salivary leakage, or preauricular hypoaesthesia [12]. However, the authors caution surgeons to advise patients preoperatively that there may be temporary complications after the surgery. We viewed the advantages of the retromandibular transparotid approach as important aspects to retain as we developed a novel modified transparotid approach using a parotid mini-incision, which we hypothesized would reduce complications and enhance aesthetic outcomes as well.

During a long period of clinical practice, we performed open reduction and internal fixation for the treatment of mandibular condyle fractures in a select group of patients using a modified transparotid approach via a parotid mini-incision and achieved satisfactory outcomes. The purpose of this propective study was to introduce the new surgical approach and to evaluate the surgical outcomes.

## Methods

### Patients

From nearly 200 patients with mandibular condylar fractures who were admitted to the Oral and Maxillofacial Surgery Department of the Ninth People's Hospital, Shanghai Jiaotong University School of Medicine between September 2006 and February 2011, a total of 31 patients with 36 mandibular condylar fractures were included in the present prospective study. Exclusion criteria were: patients with highly located mandibular condyle fractures (intraarticular fractures), those unable to tolerate anesthesia and surgery due to poor systemic condition, and those who wanted and were able to undergo conservative treatment.

All patients provided signed informed consent. Patients whose images were used for illustrative purposes related to this study report understood and agreed to permit this use of images without patient identification. The study protocol was approved by the Internal Review Board of the Ninth People's Hospital, Shanghai Jiaotong University School of Medicine.

### Surgical procedure

**Figure 1:**
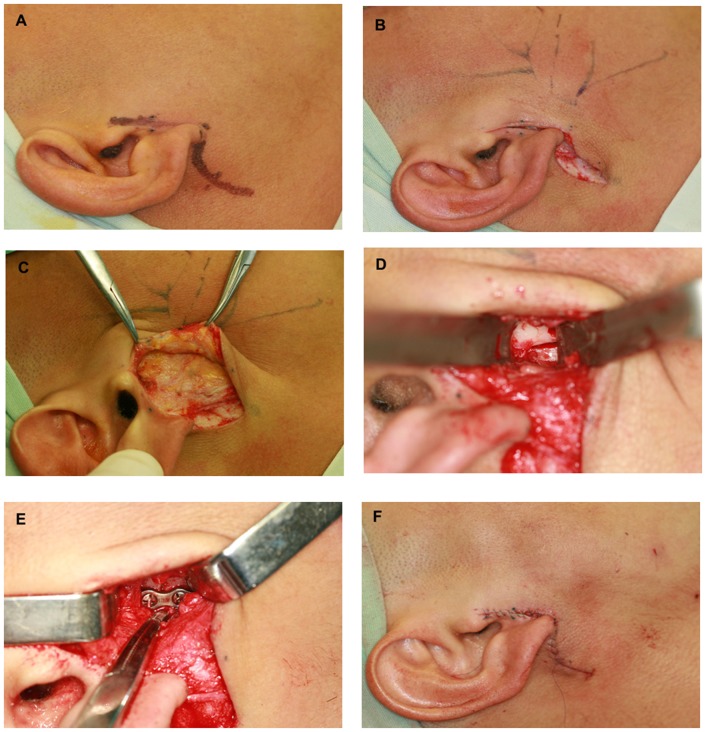
Photographs show a 25-year-old male patient undergoing open reduction and rigid internal fixation of right condylar fracture under general anaesthesia. A: Design; B: Incision; C: Flap elevation and exposure of the SMAS fascia; D: Exposure of condylar fracture; E: Rigid internal fixation; F: Suture.

All surgeries were performed by the same surgical team using the following procedure. The patient was placed in a supine position with head tilted to the contralateral side, and transnasal endotracheal intubation was carried out. The incision design required a longitudinal incision to be made along the skin on the surface of the mandibular condylar neck, and another incision was made under the earlobe according to the degree of skin laxity to release the tension (Fig. 1, A). The length of the longitudinal incision was about 1.0–1.5 cm (Fig. 1 B). After cutting open the skin and the subcutaneous tissue, the fascia masseterica were exposed. Dissection was not performed along the surface of the fascia masseterica, but it was cut open directly and the parotid gland was dissected bluntly parallel to the temporofacial branch of the facial nerve (Fig. 1 C). Then, the lateral side of the fracture site was exposed (Fig. 1 D). The periosteum was cut open and dissected with a periosteal elevator. The proximal part of the mandibular ramus was pulled down to obtain reduction of the condylar head. Two 4-hole mini titanium plates were applied to the lateral side of the mandibular condylar neck for rigid internal fixation (Fig. 1 E). The occlusal relationship was checked. A drainage tube was placed on the surface of the bone and layered suture closure was carried out for the periosteum, fascia masseterica, subcutaneous tissue and skin (Fig. 1 F).

## Results

The patients' demographic and clinical characteristics are shown in Table 1. Among all patients, 24 were males and 7 were females. The mean age was 32 years, ranging from 19 to 53 years. The 36 fractures included unilateral mandibular condyle fractures in 26 patients and bilateral mandibular condyle fractures in 5 patients. Among the 31 patients, 14 had simple mandibular condyle fractures and 17 had associated mandibular symphysis or body fractures.

**Table 1:** Patients' demographic and clinical characteristics.

Case number	Gender	Age	Unilateral Bilateral	Associated Fracture	Etiology	Occlusion*
1	F	19	Unilateral	None	RTA	Malocclusion
2	F	21	Unilateral	None	Fall	Malocclusion
3	F	52	Bilateral	Symphysis	RTA	Malocclusion
4	F	45	Unilateral	Symphysis	RTA	Malocclusion
5	F	22	Unilateral	None	Fall	Malocclusion
6	F	33	Bilateral	None	Fall	Malocclusion
7	F	32	Unilateral	Symphysis, Mandible body	RTA	Malocclusion
8	M	37	Unilateral	Symphysis	RTA	Malocclusion
9	M	49	Unilateral	Symphysis	RTA	Malocclusion
10	M	21	Unilateral	None	PV	Malocclusion
11	M	31	Unilateral	None	PV	Malocclusion
12	M	49	Unilateral	Mandible body	RTA	Malocclusion
13	M	29	Unilateral	Symphysis	RTA	Malocclusion
14	M	34	Bilateral	Symphysis, Mandible body	RTA	Malocclusion
15	M	19	Unilateral	None	PV	Malocclusion
16	M	22	Unilateral	Mandible body	RTA	Malocclusion
17	M	44	Unilateral	None	RTA	Malocclusion
18	M	26	Bilateral	Symphysis	RTA	Malocclusion
19	M	38	Unilateral	None	PV	Malocclusion
20	M	28	Unilateral	None	Fall	Malocclusion
21	M	43	Unilateral	None	PV	Malocclusion
22	M	22	Unilateral	Mandible Body	RTA	Malocclusion
23	M	35	Unilateral	Symphysis, Mandible Body	Fall	Malocclusion
24	M	29	Unilateral	Symphysis	Fall	Malocclusion
25	M	31	Unilateral	Mandible Body	Fall	Malocclusion
26	M	30	Bilateral	Symphysis	Fall	Malocclusion
27	M	39	Unilateral	None	Fall	Malocclusion
28	M	26	Unilateral	None	Fall	Malocclusion
29	M	21	Unilateral	Symphysis	RTA	Malocclusion
30	M	25	Unilateral	None	RTA	Malocclusion
31	M	53	Unilateral	Symphysis	RTA	Malocclusion

RTA: Road Traffic Accidence.

PV: Personal Violence.

All 31 patients showed malocclusion, which was one indication for open reduction and internal fixation. Patients without malocclusion were excluded from this group.

**Figure 2:**
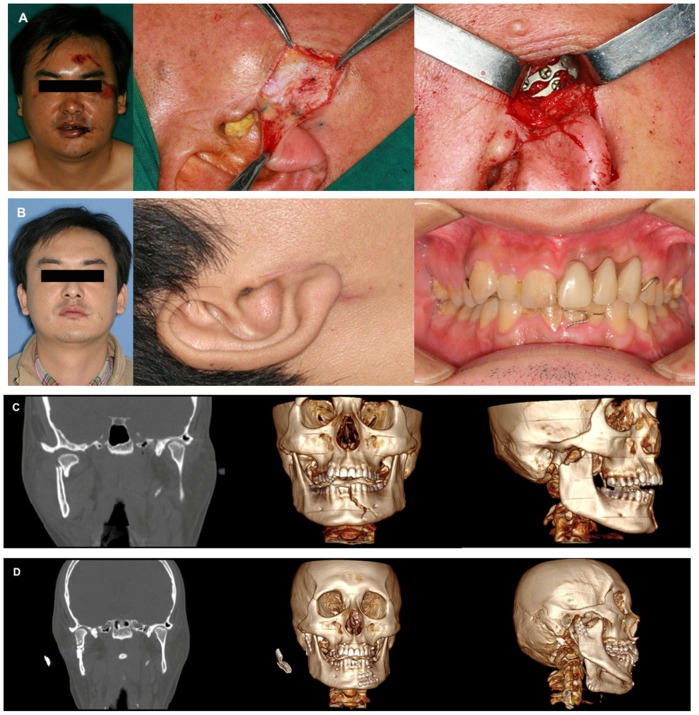
Photographs show a 33-year old male patient undergoing open reduction and rigid internal fixation of right condylar fracture 5 days after trauma. A: Pre-operative facial appearance and rigid internal fixation; B: Post-operative facial appearance and occlusion; C: Pre-operative CT scan and 3D reconstruction images; D: PT scan and 3D reconstruction images taken at follow-up.

All patients were followed up for a minimum of 3 months (range, 3 to 26 months) and postoperative results showed excellent occlusal relationships in all 31 patients (Fig. 2: A, Pre-operative appearance; B, Post-operative appearance and occlusion). No symptoms such as pain or mandibular function disturbances were reported by any patient during follow-up. No symptoms of intraoperative ipsilateral facial nerve injury such as incomplete eye closure, decreased depth of forehead wrinkles, difficulty in raising eyebrows, etc. were found in any patient. The mean degree of mouth opening was 4.0 cm (maximum 4.8 cm, minimum 3.0 cm). No mandibular deviations were noted in any patient during mouth opening. Follow-up CT within the first three months postoperatively showed complete anatomical reduction of the mandibular condyle fracture in all patients (Fig. 2: C, Pre-operative CT scan and 3D reconstruction images; D, PT scan and 3D reconstruction images at follow-up).

Postoperative complications occurred in two patients. Subauricular swelling with oozing of blood at the wound margin was seen in one patient 4 hours after surgery. During the surgical exploration for that case, a hematoma was found in the deep surface of the parotid. The hematoma was removed, the bleeding point was treated by electrical cauterization, and compression dressing was applied after placing a drainage tube a second time. The patient recovered fully. Subauricular swelling was seen in another patient one week after surgery. Results of aspiration showed pale yellow, clear liquid and possible salivary fistula was considered. Compression dressing was applied with elastic bandage after removing the salivary fluid completely and the patient recovered fully 2 weeks after treatment.

## Discussion

Over a six-year period, we treated 36 mandibular condyle fractures in 31 patients using a modified transparotid approach via a parotid mini-incision. Follow-up CT showed complete anatomical reduction of the mandibular condyle fracture in all patients. Three-month post-operative follow-up showed excellent occlusal relationships in all patients with no symptoms of intraoperative ipsilateral facial nerve injury such as incomplete eye closure, decreased depth of forehead wrinkles, or difficulty in raising eyebrows. The mean degree of mouth opening was 4.0 cm and no mandibular deviations were noted in any patient during mouth opening. The incision was well concealed in all cases and patients were satisfied with the aesthetic results of their surgeries.

Although there is no precise precedent for our procedure, studies utilizing the transparotid approach for treating mandibular condylar neck and subcondylar fractures report similar satisfactory results. A report of two cases receiving transcutaneous transparotid technique, a procedure somewhat similar to ours, emphasizes that it is possible to reach the mandibular bone going through both the parotid tissue and the masseter muscle, avoiding injuries of branches of the facial nerve [8]. The author suggests that this approach is most appropriate for placement of screws and stabilization of mandibular condyle fractures without facial nerve injury. Another study of the transparotid approach for mandibular condylar fractures reports satisfactory results in 32 patients, without complications (e.g., haematoma, bone resorption or condylar necrosis), with no mastication difficulties and with preservation of the facial nerve in all cases [13]. The authors suggest that the transparotid approach with partial parotidectomy allows good anatomical repositioning of displaced segments as a result of isolation of the facial nerve branches and removal of parotid tissue, which perfectly exposes the fracture. In our procedure, the fracture site is also directly exposed and if the facial nerve is found during dissection of the parotid tissue, it can be protected under direct vision, reducing the incidence of facial nerve injury.

Although controversy has surrounded the choice of closed reduction or open surgery for this kind of injury, scholars agree that early activity is necessary for functional recovery of the joint, and that early activity is only possible after rigid internal fixation via an open surgical approach [3]. Study results support open surgery. A prospective study of postoperative morbidity associated with open reduction and internal fixation (ORIF) of the fractured condyle found only 7 ORIF cases (14%) in 50 that had temporary weakness of the facial nerve, affecting the buccal and zygomatic branches[10]. However, no permanent weakness was found in those cases, mean recovery to normal function was 4.2 months and occlusion was satisfactory in all but one case. The authors concluded that ORIF is a safe procedure with acceptable morbidity. Therefore, except for the highly located intra-articular fractures, for which rigid internal fixation is difficult, open surgery today appears to be the mainstream approach for treating mandibular fractures at the condylar neck or subcondylar level, which are associated with significant occlusal disorders and dysfunction during mouth opening.

At present, the surgical treatment of mandibular condyle fractures may involve the use of preauricular, submandibular or retromandibular incisions, or the endoscopic-assisted intraoral approach may be applied. Each of these incisions each has its own advantages and disadvantages, and surgeons have individual preferences. The preauricular incision is 3–4 cm from the inferior border of the tragus toward the external auditory canal along the anterior part of the ear; it is more suitable for reduction of middle or high mandibular condyle fractures [5]. However, sufficient surgical exposure is difficult with this approach due to the effect of the zygomatic branch of the facial nerve, which limits the range of downward traction. Moreover, the joint capsule should also be cut open and some parts of the lateral pterygoid muscle should be excised, which may increase local injury. The submandibular incision is mainly used for middle or low mandibular condyle fractures. The incision is relatively long (1.5 to 2.0 cm) and is distant from the fracture site, but scarring is not an issue and facial nerve injury is rare [5]. However, traction of the periosteum and soft tissue limits access to the surgical field and distance increases the surgical difficulty and risk of surgical trauma. The retromandibular incision is located 1 cm inferior to the mastoid process, along the posterior margin of the ascending ramus of the mandible to the mandibular angle [14]. Although this incision can expose the posterior margin of the mandibular ramus, the surgical exposure and operation are relatively difficult for patients with a long mandibular ramus and higher mandibular condyle fractures, increasing risk of nerve and blood vessel injury [15]. Advocates for the retromandibular incision say that it is closer to the condyle process, and has better exposure of fractures of the ramus and condylar process compared to submandibular incisions [11]. A comparative study of complication rates associated with different incisions to access the fracture site of subcondylar fractures undergoing ORIF suggested that the retromandibular approaches provided a more direct visual field and nearly straight-line access for fracture fixation [16]. However, other reports indicated that this approach does not effectively protect the facial nerve; the incidence of temporary postoperative facial nerve injury is as high as 38% to 40%, and permanent facial paralysis may occur in 1% of patients [14], [15]. Nevertheless, more recent application of the modified retromandibular approach described by Tang et al. [11] resulted in only temporary facial weakness in about 8% of patients receiving the surgery. In addition to the above approaches, satisfactory results have been shown for endoscope-assisted transoral treatment of displaced bilateral condylar mandible fractures [17]. Although this technique is applied more and more widely, its main shortcoming is that it requires a relatively long learning curve and special equipment, which precludes its use in most local hospitals. The procedure followed in the present study focuses mainly on fractures located at the neck of the condyloid process and the novel use of the parotid min-incision was designed to directly expose the neck of the condyloid process, which is the major advantage of our technique.

In our experience with 36 mandibular condyle fractures in 31 patients, the smaller parotid incision provided the access needed for mandibular condylar fracture reduction and fixation, which resulted in fast healing and satisfactory concealment in our cases. Longer incisions tend to increase swelling, heal more slowly and increase risk of scarring. Evaluation of the surgical approach introduced in the present study revealed multiple advantages, as follows: 1. A small longitudinal incision 1.0–1.5 cm in length along the skin on the surface of the mandibular condylar neck is needed for this approach. Since no significant scar is left after using 6–0 Ethicon Prolene suture or intradermal suture, the approach met patients’ requirements for aesthetic effect. 2. The surgical incision is close to the fracture site and the mandibular condyle neck can be reached through the inferior part of the incision. Hence, the bone surface can be exposed without massive soft tissue dissection, which reduced the possibility of secondary injury to the peripheral soft tissue. In addition, the range of periosteal dissection is small, which ensured sufficient attachment of the periosteum after surgery, and provided sufficient blood supply to the fracture site during surgery. 3. The incision provided direct vision of the surgical site, which made it easy to perform the procedure. Meanwhile, the operator’s visual angle is vertical to the lateral side of the mandibular condyle, which allowed easy placement of the titanium plate. In addition, titanium screws can be placed perpendicular to the bone surface. Together, these characteristics made the fixation more rigid and the surgery itself simpler. 4. Using a minimally invasive small incision effectively protected the facial nerve in our cases. All cases in the current study involved the condylar neck or the subcondylar area where temporofacial branches of the facial nerve and their sub-branches are found on the surface. Therefore, when cutting open the parotid fascia, we applied a transverse or oblique incision according to the course of these nerves and used blunt dissection. The facial nerve is only a few millimeters wide and if it was found during dissection of the parotid tissue, it was protected under direct vision. These procedures were followed precisely in the present study, and no symptoms of facial nerve injury were observed in any of the 31 cases.

The limitations of our procedure must be acknowledged. First, this was a single-institution, non-randomized study with a relatively small sample. Additionally, this prospective study of 31 patients with mandibular condyle fractures was a proof-of-concept study only; it did not have a control group and did not directly compare the procedure or outcomes of the modified transparotid approach via parotid mini-incision to other surgical approaches. This limits the conclusions to observations of the studied procedure only, which in the present study showed the safety and feasibility of the procedure. Further prospective case-control study is needed to evaluate intra- and post-operative outcomes of the modified transparotid approach via parotid mini-incision compared to those of traditional surgical approaches. Clinically, because of the small incision and direct opening of the parotid, postoperative hematoma or salivary fistula may occur, as may happen with any transparotid approach. This can be handled by carefully suturing the incised parotid tissue and placing a drainage device on the surface of the mandible. In addition, when separating the parotid tissue, the temporofacial branch of the facial nerve may also be exposed, which can escape identification by surgeons not experienced in this surgery.

In conclusion, the modified transparotid approach via the parotid mini-incision is minimally invasive and achieves anatomical reduction and rigid internal fixation for mandibular condyle fractures with a simple procedure that directly exposes the fracture site. Results of this prospective case series show that the procedure is safe and feasible for treating mandibular condyle fractures and offers a short operative path and protection of the facial nerve. The use of a small, easily concealed incision provides satisfactory aesthetic outcomes.
